# Comparative Study of Two-Dimensional (2D) vs. Three-Dimensional (3D) Organotypic Kertatinocyte-Fibroblast Skin Models for *Staphylococcus aureus* (MRSA) Infection

**DOI:** 10.3390/ijms23010299

**Published:** 2021-12-28

**Authors:** Nilakshi Barua, Lin Huang, Carmen Li, Ying Yang, Mingjing Luo, Wan In Wei, Kam Tak Wong, Norman Wai Sing Lo, Kin On Kwok, Margaret Ip

**Affiliations:** 1 Department of Microbiology, Faculty of Medicine, Prince of Wales Hospital, The Chinese University of Hong Kong, Hong Kong 999077, China; nilakshibarua@cuhk.edu.hk (N.B.); 2carmen.li@cuhk.edu.hk (C.L.); claire_yang@link.cuhk.edu.hk (Y.Y.); luomingjing@cuhk.edu.hk (M.L.); kamtakwong@cuhk.edu.hk (K.T.W.); normanlo@cuhk.edu.hk (N.W.S.L.); 2 Department of Surgery, Faculty of Medicine, Prince of Wales Hospital, The Chinese University of Hong Kong, Hong Kong 999077, China; huanglin@surgery.cuhk.edu.hk; 3 Shenzhen Institute of Synthetic Biology, Shenzhen Institute of Advanced Technology (SIAT), Chinese Academy of Sciences, Shenzhen 518055, China; 4 JC School of Public Health and Primary Care, The Chinese University of Hong Kong, Hong Kong 999077, China; vivian1628@cuhk.edu.hk (W.I.W.); kkokwok@cuhk.edu.hk (K.O.K.)

**Keywords:** MRSA, skin, 3D skin model, HaCaT

## Abstract

The invasion of skin tissue by *Staphylococcus aureus* is mediated by mechanisms that involve sequential breaching of the different stratified layers of the epidermis. Induction of cell death in keratinocytes is a measure of virulence and plays a crucial role in the infection progression. We established a 3D-organotypic keratinocyte-fibroblast co-culture model to evaluate whether a 3D-skin model is more effective in elucidating the differences in the induction of cell death by Methicillin-resistant *Staphylococcus aureus* (MRSA) than in comparison to 2D-HaCaT monolayers. We investigated the difference in adhesion, internalization, and the apoptotic index in HaCaT monolayers and our 3D-skin model using six strains of MRSA representing different clonal types, namely, ST8, ST30, ST59, ST22, ST45 and ST239. All the six strains exhibited internalization in HaCaT cells. Due to cell detachment, the invasion study was limited up to two and a half hours. TUNEL assay showed no significant difference in the cell death induced by the six MRSA strains in the HaCaT cells. Our 3D-skin model provided a better insight into the interactions between the MRSA strains and the human skin during the infection establishment as we could study the infection of MRSA in our skin model up to 48 h. Immunohistochemical staining together with TUNEL assay in the 3D-skin model showed co-localization of the bacteria with the apoptotic cells demonstrating the induction of apoptosis by the bacteria and revealed the variation in bacterial transmigration among the MRSA strains. The strain representing ST59 showed maximum internalization in HaCaT cells and the maximum cell death as measured by Apoptotic index in the 3D-skin model. Our results show that 3D-skin model might be more likely to imitate the physiological response of skin to MRSA infection than 2D-HaCaT monolayer keratinocyte cultures and will enhance our understanding of the difference in pathogenesis among different MRSA strains.

## 1. Introduction

Methicillin-resistant *Staphylococcus aureus* (MRSA) is a major bacterial pathogen causing both community and healthcare infections. The emergence of community-associated (CA-MRSA) has increased the burden of staphylococcal disease worldwide, and 90% of cases of CA-MRSA infections were skin and soft-tissue infections [1]. Our systematic review on CA-MRSA carriage highlighted the increasing prevalence of this, particularly among children, in the community in the Asia-Pacific region [2]. In addition, animal-associated MRSA harboring toxins may serve as a reservoir in MRSA dissemination [3].

*Staphylococcus aureus* (SA) expresses a variety of virulence surface and secreted proteins that contribute to the colonization, initiation of infection and evasion of host immune response contributing to the pathogenesis of infection. These are well characterized among the common epidemic and sporadic clones in MRSA [4]. For example, ST30 is the predominant clonal type isolated among patients with skin and soft tissue infections in Hong Kong in comparison to USA300 which is prevalent in United States [5].

Skin is a major site of SA infection, and the pathogen penetrates the keratinocyte barrier by modulating necrosis, apoptosis, autophagy or pyroptosis [6]. In vitro studies have shown that SA can invade nonprofessional phagocytes, including fibroblasts [7], osteoblasts [8], endothelial, and epithelial cells [9]. The pathogen persists in the host cells or further escapes from the endosomes/phagosomes to disseminate into the cytoplasm leading to host cell death [10]. The complexity of the multiple virulence factors of the bacterium makes the relationship of a genotype, phenotype and infection outcome unclear [11]. In this study, we examined the variation in colonization and invasion by six strains representing the prevalent clonal types of MRSA in two in vitro infection models, namely, a two-dimensional (2D) monolayer of HaCaT cells and an organotypic three-dimensional (3D) keratinocyte fibroblast co-culture model which mimics the multi-faceted skin tissue. The bi-dimensional nature of the keratinocyte monolayer culture lacked the complex stratification and terminal differentiation of epidermal tissue [12] and failed to provide a comprehensive insight into the colonization and invasion by SA in comparison to our organotypic 3D keratinocyte-fibroblast msodel (skin model). Our skin model offers a physiologically relevant assessment of the bacterial adhesion, invasion and transmigration through the different strata of the skin model as compared to the 2D HaCaT monolayers.

## 2. Results

### 2.1. Adhesion and Internalization Assay Using HaCaT Monolayers

**Figure 1:**
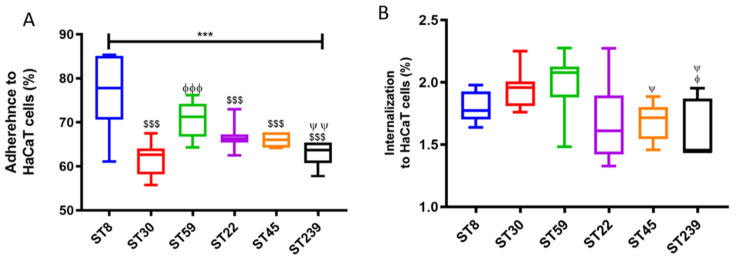
Adherence (**A**) and Internalization (**B**) of six MRSA strains representative of common ST types on HaCaT cells. Error bars represent SD of the mean, and statistical significance was calculated using (*** *p*-value < 0.001) one-way ANOVA with post hoc Tukey tests. The *p*-values are coded with respect to each strain and are derived from the comparison between the means of the two respective strains. $$$ (*p* < 0.001) shows the *p*-value of the respective strain when compared to ST8, ϕ (*p* < 0.05), and ϕϕϕ (*p* < 0.001) show significant difference when compared to ST30. ψ (*p* < 0.05) and ψψ (*p* < 0.01) depict the significant difference of the respective strain when compared to ST59. The columns with respect to the strains that do not have *p*-value symbols depict that there is no significant difference between the mean values with respect to each other. The MRSA ST8 was compared against each respective strain; ST30, ST22, ST45 and ST239 revealed *p* < 0.001 ($$$). For ST30 and ST59, *p* < 0.001 (ϕϕϕ), and for ST59 with ST239, *p* < 0.01 (ψψ), respectively. In (**B**), ST59 compared with ST45 and ST239 gave *p* < 0.05 (ψ), and for ST30 compared with ST239, *p* < 0.05 (ϕ).

The initiation of invasion of eukaryotic cells by staphylococci requires initial adherence of the bacteria to the eukaryotic cells mediated by surface protein adhesins, including microbial surface components recognizing adhesive matrix molecules (MSCRAMMs). The relative molecular and genetic variation is thought to cause variation in the virulence of the strains. Figure 1A shows the percentage of the adhered bacteria for the six MRSA strains.

The statistical difference between the adhesion of all the strains was significant as determined by the one–way ANOVA, *p* < 0.0001. The maximum adherence was demonstrated by MRSA ST8 (76.0% ± 8.6%), followed by ST59 (70.6% ± 4.1%), but there was no statistical difference between these two (*p* = 0.129, Tukey post hoc test). The percentage adherences of ST30, ST22, ST45 and ST 239 were 61.6% ± 3.7%, 62.8% ± 2.6%, 66.7% ± 2.8%, 66.1% ± 1.5%, respectively. Significant difference in percentage adherence was observed between ST59 and ST239 (*p* < 0.01). Supplementary Figure S1 shows the adhesion of the six respective MRSA strains to the HaCaT cells by Giemsa stains. The internalization assay (Figure 1B) revealed that overall, a very low percentage of the MRSA strains were internalized in the HaCaT cells, with the mean internalization of all six strains being 1.8% ± 0.2%. Tukey post hoc test revealed maximum internalization was exhibited by ST30 (1.9% ± 0.2%) and ST59 (2% ± 0.2%). Detachment of HaCaT cells was observed after 2 and half hours of the infection, which is the time taken to complete the internalization assay. Prominent detachment of the HaCaT was observed in the cells infected by ST239. Therefore, we prepared a three-dimensional organotypic keratinocyte-fibroblast co-culture model to study the colonization and invasion of keratinocytes by MRSA strains.

### 2.2. Colonization and Infection of Keratinocyte Organotypic Model

**Figure 2:**
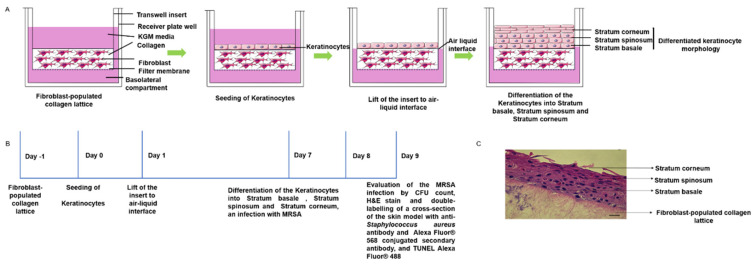
A schematic diagram showing the development of the three-dimensional human organotypic keratinocyte-fibroblast co-culture model. The figure was produced using Servier Medical Art (http://smart.servier.com/ accessed on 1 November 2021) (**A**). Timeline of the development and infection of the organotypic keratinocyte-fibroblast co-culture model (**B**). H&E staining of the cultured organotypic keratinocyte-fibroblast co-culture model. The image represents Day 7 culture in air-liquid interface. Scale Bar shows 20 µm (**C**).

The skin model was prepared (Figure 2A,C) and infected by MRSA strains at day seven of air–liquid interface culture (Figure 2B).

**Figure 3:**
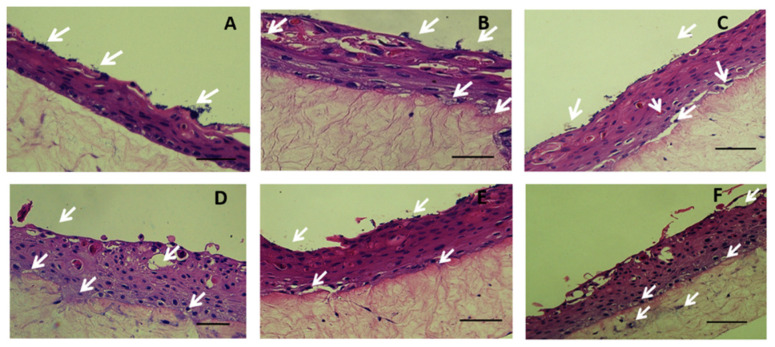
H&E stain of histological section of skin model at 48 h after inoculation with MRSA strain ST8 (**A**), ST30 (**B**), ST59 (**C**), ST22 (**D**), ST45 (**E**) and ST239 (**F**), respectively. White arrows show microcolonies of bacteria. Scale Bar depicts 50 µm.

After 48 h of infection, exfoliation of the strata corneum and strata spinosum of the skin model was observed by the MRSA strain ST8 (Figure 3A) and the ST30 (Figure 3B) shown in white arrows. The MRSA strain ST59 adhered to the stratum corneum and formed microcolonies. We observed damage of stratum basale at 48 h (Figure 3C). The MRSA strain ST22 adhered to the stratum corneum of the skin model; exfoliation of the stratum corneum was observed (Figure 3D). The MRSA strain ST45 exhibited exfoliation of the stratum corneum damage to the stratum basale at 48h (Figure 3E). However, ST45 did not exhibit damage of stratum spinosum. Damage to the stratum corneum upon infection by the MRSA strain ST239 has been observed (Figure 3F).

**Figure 4:**
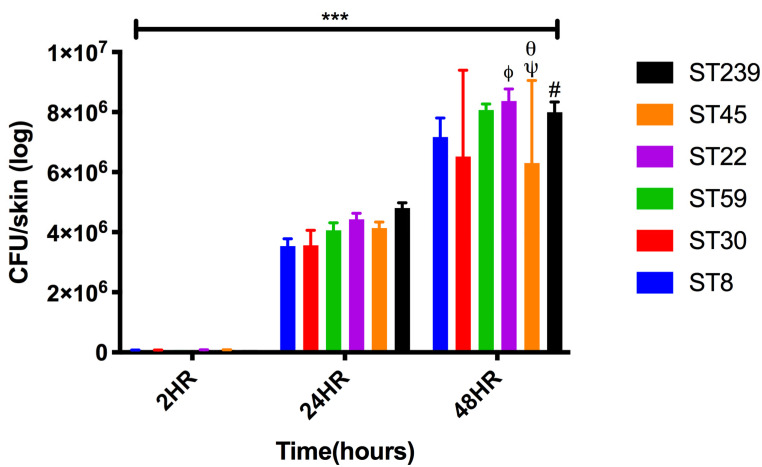
Enumeration of adherent and internalized bacteria of MRSA strains representative of common ST types on skin model. Error bars represent SD of the mean, statistical significance was calculated using two-way ANOVA (*** *p* < 0.001) and Tukey post hoc test. The *p*-values are coded with respect to each strain and are derived from the comparison between the means of the two respective strains. *p*-values were significant at *p* < 0.05 when compared to ST30 (ϕ), ST59 (ψ), ST22 (θ), and ST45 (#).

The CFU enumeration of the bacteria per skin model (Figure 4) shows one log increase at 24 h and 48 h when compared to the time point of 2 h after inoculation. At 48 h, maximum adhesion and internalization of the bacteria were observed in case of ST22 (8.4 × 10^6^ ± 4.1 × 10^5^) followed by ST59 (8.1 × 10^6^ ± 2.1 × 10^5^), and ST239 (8.0 × 10^6^ ± 3.4 × 10^5^).

### 2.3. Invasion and Cell Death Modulation by the MRSA Clonal Types

**Figure 5:**
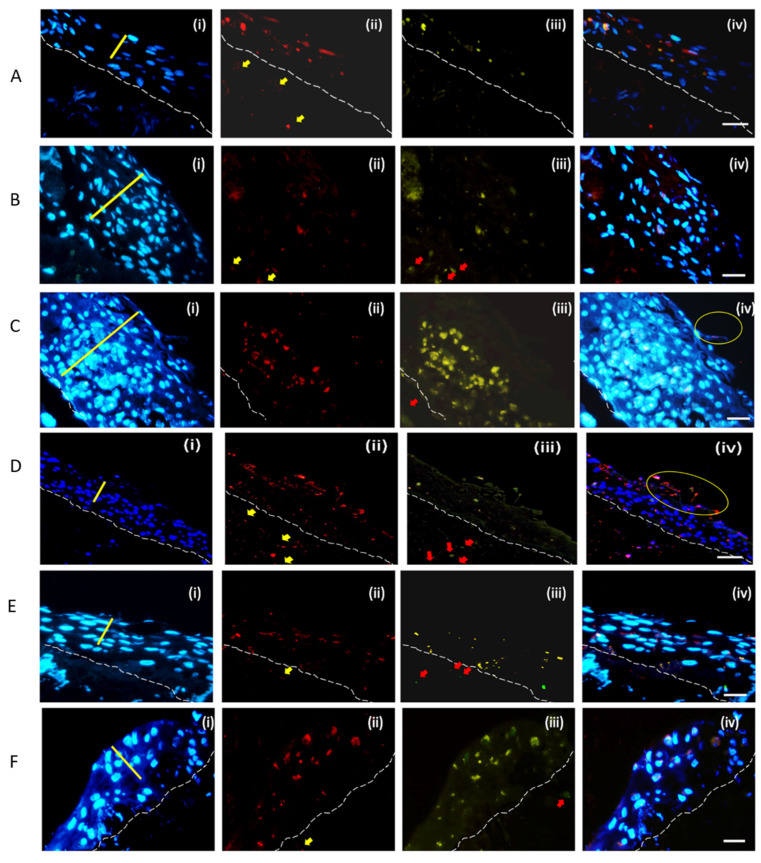
Images of a double-labelling of a cross-section of the skin model after 48 h infection with MRSA strain ST8 (**A**), ST30 (**B**), ST59 (**C**), ST22 (**D**), ST45 (**E**) and ST239 (**F**) using (i) DNA-binding Hoecsht stain (blue) to reveal the nuclei of the keratinocytes at the strata basale and spinosum, (ii) bacteria detected using anti-*S. aureus* antibody and Alexa Fluor^®^ 568 conjugated secondary antibody, (iii) the Click-iT^®^ TUNEL Alexa Fluor^®^ 488 cell. (iv) is an overlay of the emission signals. It shows co-localization of bacteria and apoptosis/DNA damage in keratinocytes in strata spinosum. White hashed line demarcates the dermal epidermal boundary between the stratum basale and the collagen gel populated with fibroblasts. The yellow line in (i) shows the stratum spinosum and applies across (i) to (iv). The yellow arrows in (ii) show the bacteria in the collagen gel. The yellow circles in (iv) show the exfoliation of the skin model. Scale Bar shows 50 µm and applies to (i) to (iv).

**Figure 6:**
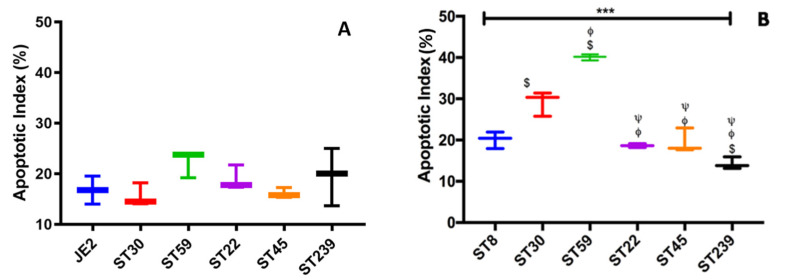
Quantification of the apoptotic index (%) (**A**) in HaCaT cells 2 and half hour post infection and (**B**) in the skin model 48 h post infection with six MRSA strains representative of the common ST types. Error bars represent SD of the mean; statistical significance was calculated using one-way ANOVA (*** *p* < 0.001) and Tukey post hoc test. The p values are coded with respect to each strain and are derived from the comparison between the means of the two respective strains. Statistical significance was obtained at *p* < 0.05 when the strain was compared to ST8 ($), ST30 (ϕ) and ST59 (ψ), respectively.

The Click-iT TUNEL Alexa Fluor^®^ 488 Imaging Assay was used to study the cell death modulation by the different MRSA strains. The manufactures instruction manual was followed. A TUNEL assay revealed apoptotic-positive keratinocytes marked by green staining induced by the infection of the six different clonal types MRSA in HaCaT cells (Supplementary Figure S2). Figure 5 shows the double-labelling assay of the respective cross section of the skin model after 48 h of MRSA infection. The bacterial detection was by immunohistochemical staining using *anti-S. aureus* antibody and Alexa Fluor^®^ 568 (red) conjugated secondary antibody. The apoptosis was detected by Click-iT TUNEL Alexa Fluor^®^ 488 (green). The blue Hoechst stain marked the DNA. The study provided an insight in the interactions between the different MRSA strains and the human skin during the establishment of the infection. Dissemination of ST8 through the stratified epidermal keratinocyte to the fibroblast populated collagen lattice was observed (Figure 5A(ii)). ST8 induced cell death on the stratum basale and stratum spinosum (Figure 5A(iii)). The ST30 dissemination and cell death induced was observed to be dispersed in all the three strata of keratinocytes Induction of cell death of fibroblasts by the ST30 was observed (Figure 5B(iii)). ST59 disseminated throughout the three layers of the skin model and induced cell death. Cell death was observed to be concentrated more in the stratum spinosum and stratum basale. Death of the fibroblasts in the collagen gel was also observed (Figure 5C(iii)). The yellow circle in Figure 5C(iv) shows the bound bacteria as well as exfoliation of the skin model. Dissemination of the ST22 was seen in all the strata of the keratinocytes and in the collagen lattice (Figure 5D(ii)). Induction of cell death by ST22 (Figure 5D(iii)) was observed in keratinocytes and the fibroblast. The yellow circle of Figure 5D(iv) shows the exfoliation of the keratinocytes with the ST22 bacteria bound to the keratinocytes. The cell death is lower when compared to the dissemination of the ST22 throughout the skin model. Figure 6B also shows that ST22 has the lowest apoptotic index. ST45 dissemination in all the strata of the skin model can be seen in Figure 5E(ii). Cell death was observed in keratinocytes as well as in the fibroblasts. ST239 (Figure 5F(ii)) disseminated in to all the strata of the skin model and induced cell death in keratinocytes and the fibroblasts.

Apoptotic Index was calculated using the Formula in equation 3 for the HaCaT cells as well as the skin model. One-way ANOVA and Tukey post hoc test showed no significant difference (*p* < 0.05) in the Apoptotic index among the six MRSA strains tested (Figure 6A) in HaCaT monolayers at 2 and a half hours of infection. The mean apoptotic index for all the six strains was observed to be 18.2% ± 2.5%. The highest apoptotic index was observed to be of ST59 (22.3% ± 2.7%), and the lowest apoptosis was induced by ST30 (15.6% ± 2.5%). The results presented for the induction of apoptosis in HaCaT Keratinocytes are after the inoculation of 2 and a half hours of infection as the cells from the monolayers started to detach after 2 and a half hours of bacterial inoculation. In the 3D-skin model, one-way ANOVA of apoptotic index at 48 h of infection showed significant difference with *p*-value *** *p* < 0.001) (Figure 6B). The mean apoptotic index for all the strains was 23.6% ± 9.4%, with the range from 14.3% ± 1.5% (ST239) to 40.1% ± 0.7% (ST59). Tukey post hoc test showed that ST59 exhibited the highest Apoptosis, with a *p*-value < 0.001 with respect to each strain tested. The lowest apoptosis was observed in ST239. 

## 3. Discussion

Adherence to a specific substrate plays a critical role in colonization and the initiation of an infection [13]. The microbial surface components recognizing adhesive matrix molecules (MSCRAMMs) expressed by *S. aureus* recognize and bind to the host cells through the extracellular matrix molecules such as fibronectin, fibrinogen, and collagen, implicating that adherence to these human extracellular matrix proteins plays a major role in the pathogenesis of *S. aureus* [14]. The difference in the expression of these MSCRAMMs by a different sequence type of MRSA may play a key role in determining the propensity of the infection. The direct interaction between fibronectin-binding proteins (FnBPs) and the heat shock protein (HSP-60) expressed by epithelial cells plays a major role in invasion [15]. Fibrinogen plays a prominent role in host antimicrobial defense, but *S. aureus* has evolved to produce Fibrinogen-binding proteins [16] which enables it to interact and hijack the host coagulation system to promote pathogenesis [17]. We investigated the adhesion of the MRSA strains to solid phase fibrinogen and fibronectin (Supporting Data S1, Supplementary Figure S3). Our data show that there is no apparent difference in binding to the solid phase fibrinogen between the MRSA strains representing ST59 and ST45, and both exhibited the maximum binding. The lowest binding to the solid phase fibrinogen was exhibited by ST8 and ST30 with no significant differences between them. In case of the solid phase fibronectin binding assay, no significant statistical difference was observed among the binding of the MRSA strains to fibronectin except for the ST239. The MRSA ST239 strain had the lowest binding to fibronectin with significant difference compared to ST8, ST30, ST59 and ST22. For a better understanding of the nascent field of the unique host–pathogen interactions of these different MRSA strains, we studied the adhesion and internalization of these MRSA strains by HaCaT monolayers.

Internalization and the ability to survive within host cells like keratinocytes [18] may decide the outcome of the *S. aureus* infection by controlling the dispersal of the bacteria, causing chronic infections or deeper tissue infections and dissemination to sterile sites [19] Upon internalization by the host cells, *S. aureus* modulates specific cellular responses in vivo, which ultimately influences the course of infection [20]. Bur et al., 2013 [21], reported that adhesion as well as internalization of *S. aureus* was more effectively to/into HaCaT cells than in NHEK cells, and hence, we selected HaCaT cells for our studies. We found that the strains ST8 and ST59 did not show significant difference in adherence to HaCaT cells. The adherence of the MRSA strains ST30, ST22, ST45 and ST 239 to HaCaT cells was similar except for significant differences between ST59 and ST239. HaCaT cells being non-professional phagocytes internalized an extremely low percentage of the MRSA.

Two-dimensional (2D) monolayer of keratinocytes have provided a functional perception of the *S. aureus* pathogenesis including many facets which include bacterial attachment, invasion and the innate immune response in by keratinocytes [22,23]. However, we observed that the bi-dimensional nature of the HaCaT monolayer could not provide us the platform to study the differences in the stimulation of cell death by the MRSA strains for a longer period of time. The detachment of keratinocytes from the culture wells limited the TUNEL assay to 2 and half hours, and no significant difference was observed in the Apoptotic Index among the six strains tested. 

The use of rodent models to study *S. aureus* infection makes interpretation of data complex due to differences in the histology and immunology between human skin and rodent skin [24]. Additional drawbacks of the use of rodent skin models include the requirement of inoculation of bacteria by mechanical disruption of the rodent skin [25,26,27] which does not mimic the colonization niche of *S. aureus* within an intact tissue and does not address the pathogenesis of soft tissue invasion or systemic infection by asymptomatic colonizing *S. aureus*. Human skin models provide the niche to study the host–*S. aureus* interactions and can be widely categorized into the ex vivo human skin explant cultures and the regenerated 3D organotypic skin models. The human skin explants have the advantage of possessing the conserved epidermis and dermis structure including the skin appendages and being viable for up to 14 days in cell-culture medium. However, these models are limited to the availability of the skin explants like foreskin, cadaveric or surgical tissues and also limited experimental manipulation of host genetics. The regenerated organotypic 3D-skin models are constructed using primary human or immortalized human keratinocytes with the flexibility of incorporation of melanocytes, Langerhans cells, endothelial and nervous cells [28,29,30]. Organotypic 3D-skin models constructed using HaCaT cells make them more reproducible as they do not contain the genetic variability arising due to the primary keratinocytes from different donors. However, this is overridden by the lack of differentiation and stratification of the HaCaT 3D-skin model in comparison to the primary keratinocyte 3D-skin model [31]. 3D bioprinting presents a fully automated, scalable and advanced platform that facilitates the development of skin constructs. The simultaneous and precise deposition of multiple types of cells and biomaterials facilitates the development of 3D in vitro pigmented human skin constructs, skin model with sweat glands and hair follicles [32,33,34,35,36]. We have constructed a 3D-skin model to study the colonization and invasion of keratinocytes by MRSA strains which provides the platform of differentiated stratified keratinocyte layers to study the invasion of the epidermis. The use of human primary keratinocytes and the fibroblast in our skin model allows studying the bacterial colonization in a physiologically relevant model in comparison to rodent infection model. The constraint of our model is the genetic variability that will arise due to heterogeneous donors of primary keratinocytes and fibroblasts. Further improvement of our model could be done by incorporation of melanocytes, nervous cells and Langerhans cells.

We followed the colonization and internalization of the MRSA strains in the skin model up to 48 h. Haematoxylin and Eosin staining of the FFPE 5 µm tissue cross sections revealed the transmigration of the bacteria in the skin models as well as the differences in the damage induced to the stratified layers by the respective MRSA strains. We investigated the invasion of the skin model by the different strain of MRSA along with the cell death by the double staining with anti-*Staphylococcus aureus* antibody and the TUNEL assay after 48 h of infection. Cell death of endothelial host cells has been reported to be induced by *S. aureus* by apoptosis. Menzies and Kourtevahave reported Apoptosis within 1hr of the infection just like us and is dependent on the internalization of the live bacteria and not on the adhesion of the bacteria [37] in HaCaT cells. The localization of the bacteria correlated with apoptosis in the skin model. In this context, maximum internalization is exhibited by ST59 which correlates to the maximum cell death induced in HaCaT cells as well as the Keratinocyte organotypic culture. 

Our 3D skin model provides a convenient platform for studying *S. aureus* invasion of the multifaceted skin tissue. The presence and the expression of the armory of virulence factors of these MRSA strains play a significant role in the invasion of the skin by modulating the cell death of the keratinocytes. Further experimental investigations will provide discernment of the interactions between the bacteria and the skin tissue.

## 4. Materials and Methods

### 4.1. Bacterial Strains, HaCaT Cells, Primary Human Cells and Reagents

Clinical strains of MRSA, representing globally important clonal types, namely, ST30, ST59, ST22, ST45 and ST239 and ST8 (JE2 USA300) were used in our study. The strains were molecularly characterized as in our previous reports [4,38]. The sequence types and the genotypes with characteristic virulence factors of MRSA are provided in the Supplementary Table S1. The MRSA strains were cultured in tryptic soy broth (TSB, Oxoid, Hampshire, UK) or brain heart infusion (BHI, Oxoid, Hampshire, UK) broth at 37 °C, and a 1% broth was grown to an OD_595nm_ of 0.4 and harvested at midlog phase. HaCaT cells were seeded at 5 × 10^5^ cells per well, and cultured in Dulbecco’s Modified Eagle’s medium (DMEM, Gibco, Grand Island, NY, USA) supplemented with 10% FBS (Gibco, Grand Island, NY, USA), at 37 °C, 5% CO_2_ for 24 h until confluent and allowed to mature for another 24 h before bacterial inoculation. The human keratinocytes and fibroblasts were obtained from discarded surgical foreskins from a single donor and stored in liquid nitrogen until use. Ethical approval was obtained from the respective Institutional Research Ethics Committee (CRE-2004.433 and CRE-2006.434). Unless otherwise stated, all the other reagents were obtained from Sigma (St. Louis, MO, USA).

### 4.2. Adhesion and Internalization Assays Using HaCaT Monolayers

The adhesion and internalization assays were performed as previously described [21], with slight modifications. The matured HaCaT cells were maintained in DMEM supplemented with 1% FBS for 1 h to avoid interference from serum components before bacterial infection. Bacterial cells were grown to OD_595nm_ = 0.4 and were harvested by centrifugation at 3000× *g* for 10 min and washed with phosphate buffered saline (PBS). The bacterial cells were suspended in DMEM medium with 1% FBS and then added to keratinocytes at a MOI of 100 and co-cultivated with HaCaT cells at 37 °C, 5% CO_2_ for 90 min. The MOI of 100 was selected for the study because only 4% of bacteria have been reported to be internalized by keratinocytes after 48 h of incubation in comparison to macrophages, which are reported to internalize 25% of the bacteria within 15 min of inoculation [39]. Thereafter, the keratinocytes were detached using 0.5% Trypsin (Gibco, Grand Island, NY, USA) after removal of any unbound bacteria by PBS. The detached cells were lysed by incubation with 500 µL 0.5% Triton X-100 (Sigma, St. Louis, MO, USA) per well for 15 min. Colony forming units (CFU) were enumerated by serial dilution and plating of the lysates onto horse blood agar plates and incubation overnight at 37 °C. The percentage of adherence of SA to the HaCaT monolayers was calculated using the following Equation (1)

(1) $$Adherence\;of\;S.\;aureus\;to\;HaCaT\;cells\;in\;\%=\frac{Number\;of\;cfu\;recovered\;per\;well}{Number\;of\;cfu\;inoculated\;per\;well}\times 100$$

The internalization assay was performed similar to adhesion assay. After removal of the non-adherent bacterial cells, the HaCaT cells were incubated with DMEM media supplemented with 100 µg/mL gentamycin,10 µ g/mL lysostaphin and 1% FBS for 1 h at 37 °C to kill extracellular bacteria. The antibiotics were removed with PBS, and the cells were then enumerated as mentioned in the adhesion assay. The internalization of the SA to the HaCaT monolayers was calculated using the Equation (2) which is as follows:

(2) $$Internalization\;of\;S.\;aureus\;to\;HaCaT\;cells\;in\;\%=\frac{Number\;of\;cfu\;recovered\;per\;well}{Number\;of\;cfu\;inoculated\;per\;well}\times 100$$

### 4.3. D Skin Model: Keratinocyte Organotypic Culture on Collagen Gel Incorporated with Fibroblast

In order to evaluate the staphylococcal colonization and the transition to invasive infection, we established a skin model using primary fibroblasts and primary keratinocytes in Corning Transwell (Corning, Kennebunk, ME, USA) polyester membrane cell culture 12 mm inserts with 0.4 µm pores according to our previous study [40] with slight modifications. Frozen stocks of keratinocytes and fibroblasts were used. Collagen gel was prepared using rat-tail type-I collagen (ibidi, Gräfelfing, Munich) to a final concentration of 3 mg/mL in 1.2 X DMEM neutralized with 5 M NaOH to a final concentration of 3 × 10^−3^ M. The fibroblast cells were mixed with the collagen gel to obtain a final cell density of 1 × 10^5^ cells/mL in order to generate the fibroblast-populated collagen lattice (FPCL), and 600 μL of this mixture was loaded into each transwell. The FPCL was polymerized at 37 °C, 5% CO_2_ for 2 h and equilibrated in Keratinocyte growth medium (KGM) overnight. The KGM is composed of 1:3 mixture of Ham’s F12 (Gibco, Grand Island, NY, USA) and DMEM (Gibco, Grand Island, NY, USA), 10% FBS (Gibco, Grand Island, NY, USA), 5 μg/mL insulin (Sigma, St. Louis, MO, USA), 1.8 × 10^−4^ M adenine sulfate (Sigma, St. Louis, MO, USA), 10^−10^ M cholera toxin (Sigma, St. Louis, MO, USA), 0.4 μg/mL hydrocortisone (Sigma, St. Louis, MO, USA), 1ng/mL EGF (Gibco, Grand Island, NY, USA) and 0.1% BSA (Sigma, St. Louis, MO, USA). After 24 h equilibration of the FPCL with KGM, the Keratinocyte organotypic culture was generated by seeding of 2 × 10^5^ keratinocytes suspended in 200 µL KGM on top of the FPCL in each transwell. The attachment of the keratinocytes to the FPCL was allowed for 24 h by incubating the transwells at 37 °C in 5% CO_2_, and then, the insert was lifted to an air–liquid interface. The model was cultured for an additional 1 week with KGM being replaced every 2 days. All experiments were performed in triplicates on 1-week air-exposed cultures.

### 4.4. MRSA Colonization on 3D-Skin Model

Bacterial suspension of 2 × 10^7^ CFU in 100 µL of KGM medium was added to each transwell and incubated for 1 h at 37 °C. The non-adherent/loosely adherent bacteria were removed from the culture by aspiration at 2, 24 and 48 h of infection and collected in 1mL of PBS. The 3D co-culture from each transwell was harvested at the defined timepoint and dissected in two equal halves. One-half was used for histological studies, and the other half was homogenized in PBS using a glass Potter–Elvehjem tissue homogenizer (Sigma, Allentown, PA, USA). Bacterial counts were enumerated by serial dilution and plating for CFU counts [41].

### 4.5. Histology

One half of each skin model was fixed in 4% (*v/v*) formaldehyde, dehydrated, and then embedded in paraffin. The paraffin blocks were cut into 5 μM sections followed by deparaffinization and rehydration for Haematoxylin and eosin (H&E) staining and TUNEL assay.

### 4.6. Assessment of Bacterial Dissemination and Cell Death Modulation in the Skin Model

TUNEL Assay was performed to analyze the cell death induced by the bacterial cells. The assay was performed following the manufacturer’s instructions of Click-iT Plus TUNEL Assay for in situ apoptosis detection with Alexa Fluor dyes (Life Technologies, Burlington, ONT, Canada). HaCaT cells were harvested at a timepoint of 150 min after bacterial incubation and after washing steps for adhesion assay. Cells were fixed using 4% paraformaldehyde in PBS and permeabilized using 0.25% TritonX-100. In case of the formalin fixed paraffin embedded tissue, the tissue sections were deparaffinized with xylene and rehydrated. The sections were fixed with 4% paraformaldehyde and treated with Proteinase K. The end labelling was performed by catalytic addition of dUTP by TdT 60 min at 37 °C. The dUTP utilized contains a small, bio-orthogonal functional group, and detection was performed using a click reaction between an azide and alkyne catalyzed by copper (I). Bacteria were labeled with anti-*Staphylococcus aureus* antibody (Abcam, Cambridge, UK) at 1:250 dilution in PBS on the histological sections revealing various strata of the skin. Samples were incubated overnight at 4 °C, protected from light. The secondary antibody solution of Goat Anti-Rabbit IgG H&L conjugated with Alexa Fluor 568 (Abcam, Cambridge, UK) was added at the concentration of 2 µg/mL and incubated for 1 h at room temperature. The DNA stain Hoechst (Sigma, St. Louis, MO, USA) was used as a counter stain.

### 4.7. Apoptotic Index

The whole area of the sections was scanned for apoptotic keratinocytes. The AI was then calculated using the following Equation (3)

(3) $$AI=\frac{Number\;of\;apoptotic\;keratinocytes\;persection}{Total\;number\;of\;keratinocytes\;per\;section}\times 100$$

### 4.8. Statistical Analysis

One-way ANOVA was applied with Tukey post hoc test to compare the mean of each strain with the mean of every other stain in the test. The errors bars show the standard deviation. The *p*-values are coded with respect to each strain and are derived from the comparison between the means of the two respective strains. Each experiment was performed in triplicates and repeated thrice. The experiments on the keratinocyte organotypic culture were performed in triplicate for each MRSA strain and each time point.

## Supplementary Materials

The following are available online at https://www.mdpi.com/article/10.3390/ijms23010299/s1, Figure S1: Microscopic images (1000×) of Giemsa stained HaCaT keratinocytes adhered MRSA strain ST8 (A), ST30 (B), ST59(C), ST22 (D), ST45 (E) and ST239 (F) respectively. The HaCaT cells were cultured in Nunc Lab-Tek Chamber Slide system with DMEM supplemented with 10% FBS at 37 °C, 5% CO_2_ for 24 h until confluence. The Adhesion Assay was performed, and the slides were then fixed in 100% methanol for 30 min and stained for 30 min with a freshly prepared solution of 10% Giemsa stain. The slides were rinsed off in water, dried and were mounted to view under oil immersion with a 100× objective. Scale Bar shows 10 µm and applies to (A) to (F), Figure S2: A TUNEL assay revealed apoptotic-positive HaCaT cells induced by the infection of the different MRSAstrains. (i) the Click-iT^®^ TUNEL Alexa Fluor^®^ 488 cells, (ii) Hoecsht stain for nuclei (iii) the overlay of the two emission signals from TUNEL positive tissue. (a) Control HaCaT keratinocytes without MRSA infection and (b) ST8, (c) ST30, (d) ST59, (e) ST22, (f) ST45 and (g) ST239. Scale Bar shows 20 µm and applies to (i) to (iii), Figure S3: Adherence of six MRSA strains representative of common ST types on solid phase coated with (A) Fibrinogen and (B) Fibronectin. Error bars represent SD of the mean, statistical significance was calculated using two-way ANOVA (*** *p* < 0.001) and Tukey post-hoc test. The MRSA ST8 was compared against each respective strain ST59, ST45 and ST239 and revealed *p* < 0.001 ($$$); with ST22, *p* < 0.01 ($$) in (A), while ST8 and ST239 in (B) revealed *p* < 0.001 ($$$). Statistical significance for ST30 with respect to ST59 and ST45 was *p* < 0.001 (ϕϕϕ) in (A) and with ST239 in (B), *p* < 0.05 (ϕ). A *p*-value < 0.001 (ψψψ) was obtained for ST59 with respect to ST22 and ST239 respectively in (A). For ST45 with ST239, *p* < 0.001 (###) in (A), and in (B), *p* < 0.05 (#). In (B), ST22 was compared with ST239, *p* < 0.05 (θ), Table S1: The clonal complex, *spa* type, MLST types, SCC*mec* types and virulence genes in six MRSA strains used in this study, Supporting Data S1: Solid Phase Fibrinogen and Fibronectin Binding Assay.
