# Supplementary material for: Comparative Study of Two-Dimensional (2D) vs. Three-Dimensional (3D) Organotypic Kertatinocyte-Fibroblast Skin Models for Staphylococcus aureus (MRSA) Infection
Source: Int J Mol Sci. 2021 Dec 28;23(1):299. doi: 10.3390/ijms23010299 (PMC8745520; doi:10.3390/ijms23010299)
Supplement: Supplementary file 1 [file ijms-23-00299-s001.zip › ijms-1508602-supplementary files/Supplemenatry Figure S1-S3.pdf]

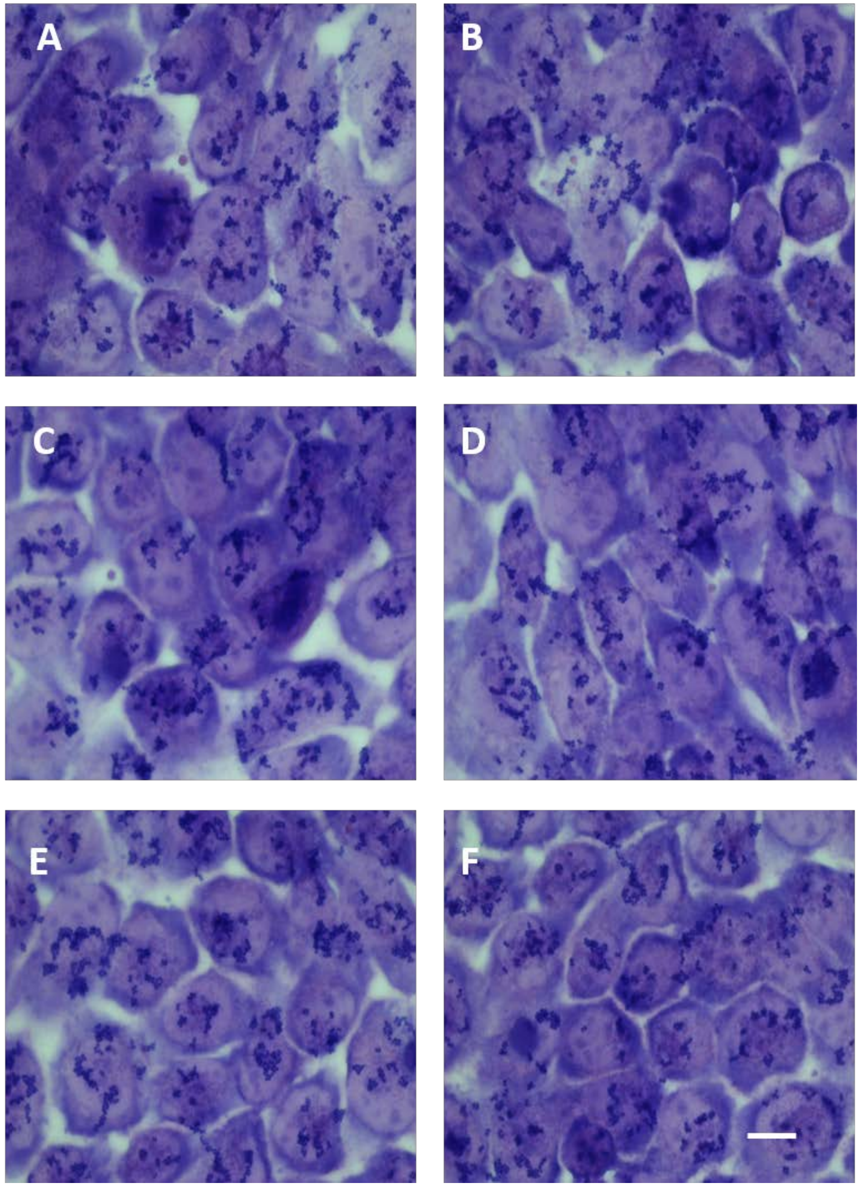

Supplementary Figure S1. Microscopic images (1000x) of Giemsa stained HaCaT keratinocytes adhered MRSA strain ST8 (A), ST30 (B), ST59(C), ST22 (D), ST45 (E) and ST239 (F) respectively. The HaCaT cells were cultured in Nunc Lab-Tek Chamber Slide system with DMEM supplemented with 10% FBS at 37 ° C, 5% CO2 for 24 hrs until confluence. The Adhesion Assay was performed, and the slides were then fixed in 100% methanol for 30 minutes and stained for 30 minutes with a freshly prepared solution of 10% Giemsa stain. The slides were rinsed off in water, dried and were mounted to view under oil immersion with a 100x objective. Scale Bar shows 10μm and applies to (A) to (F).

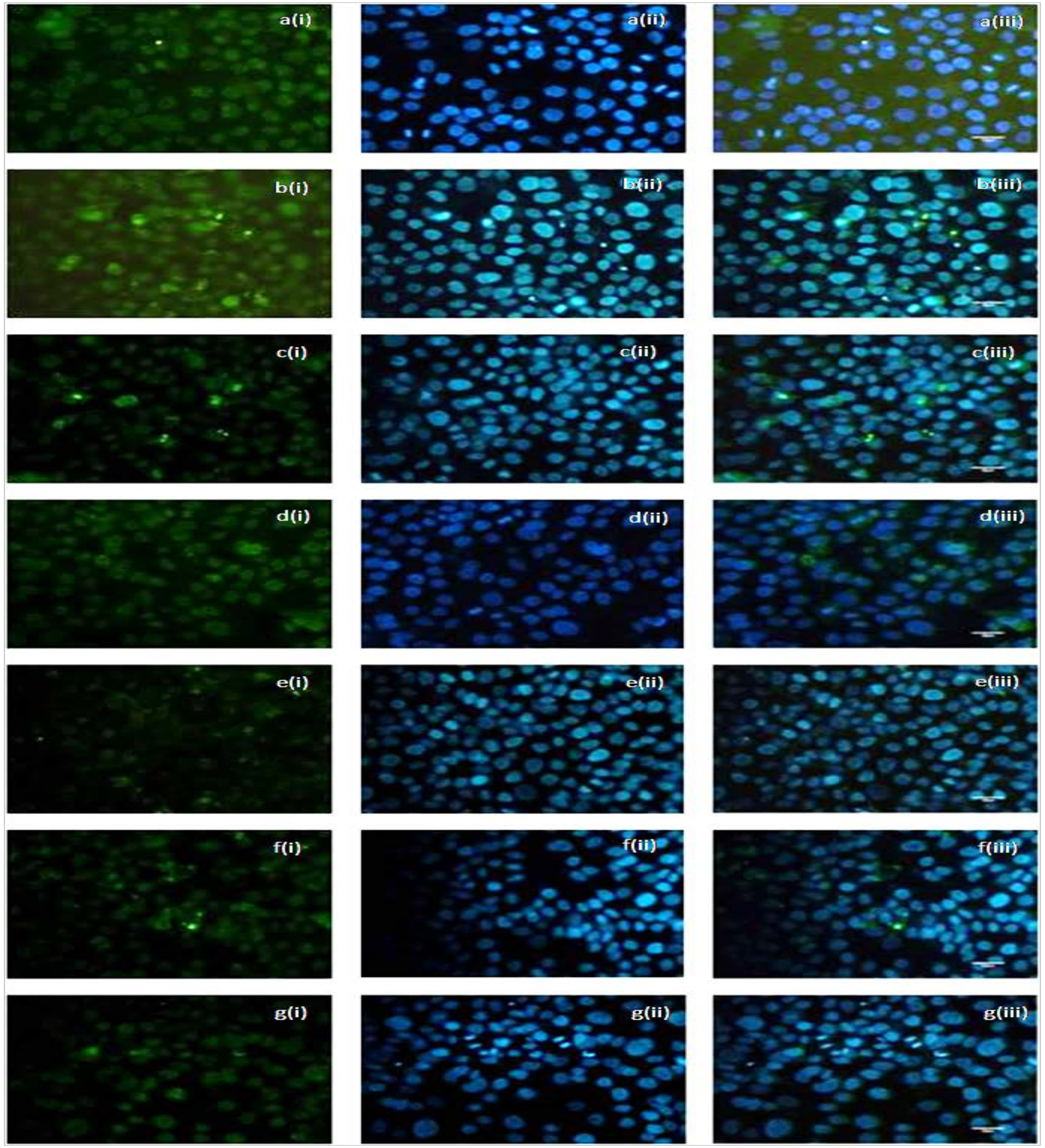

Supplementary Figure S2. A TUNEL assay revealed apoptotic-positive HaCaT cells induced by the infection of the different MRSAstrains. i) the Click-iT® TUNEL Alexa Fluor® 488 cells, ii) Hoecsht stain for nuclei iii) the overlay of the two emission signals from TUNEL positive tissue. (a) Control HaCaT keratinocytes without MRSA infection and (b) ST8, (c) ST30, (d) ST59, (e) ST22, (f) ST45 and (g) ST239. Scale Bar shows 20µm and applies to (i) to (iii).

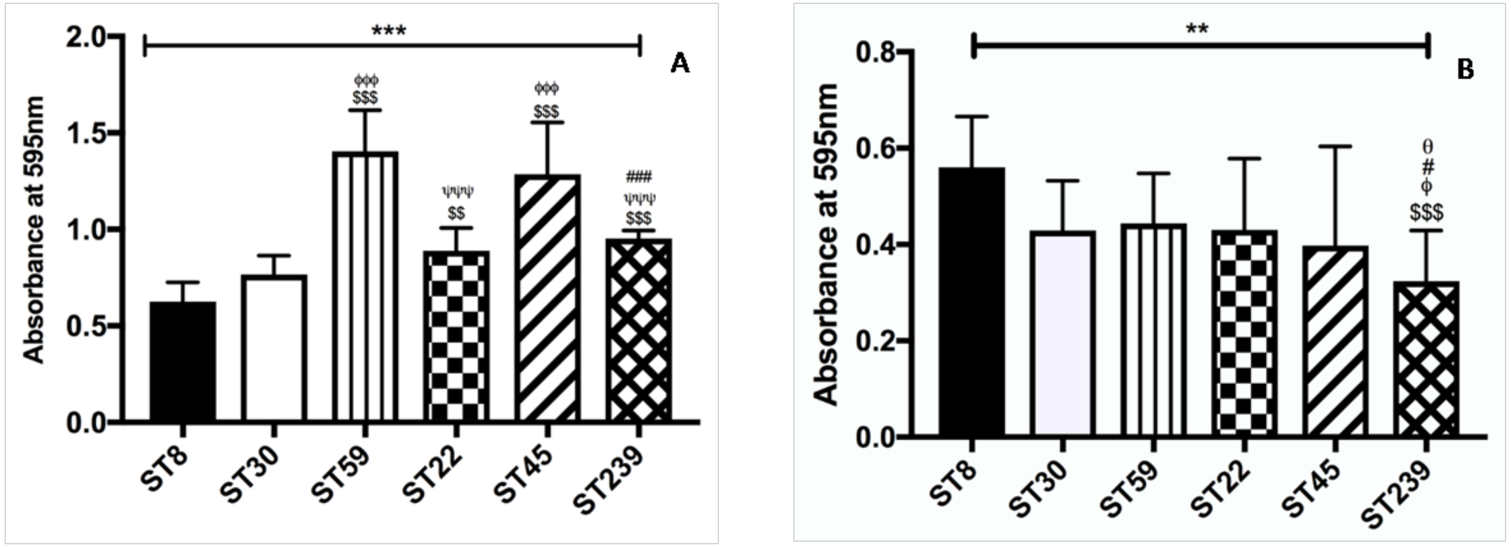

Supplementary Figure S3. Adherence of six MRSA strains representative of common ST types on solid phase coated with (A) Fibrinogen and (B) Fibronectin. Error bars represent SD of the mean, statistical significance was calculated using two-way ANOVA (*$p < 0.001$) and Tukey post-hoc test. The MRSA ST8 was compared against each respective strain ST59, ST45 and ST239 and revealed $p < 0.001$ ($$$); with ST22, $p < 0.01$ ($$) in (A), while ST8 and ST239 in (B) revealed $p < 0.001$ ($$$). Statistical significance for ST30 with respect to ST59 and ST45 was $p < 0.001$ (ϕϕϕ) in (A) and with ST239 in (B), $p < 0.05$ (ϕ). A *p*-value < 0.001 (ψψψ) was obtained for ST59 with respect to ST22 and ST239 respectively in (A). For ST45 with ST239, $p < 0.001$ (###) in (A), and in (B), $p < 0.05$ (#). In (B), ST22 was compared with ST239, $p < 0.05$ (θ).**
