# Supplementary material for: Comparative Study of Two-Dimensional (2D) vs. Three-Dimensional (3D) Organotypic Kertatinocyte-Fibroblast Skin Models for Staphylococcus aureus (MRSA) Infection
Source: Int J Mol Sci. 2021 Dec 28;23(1):299. doi: 10.3390/ijms23010299 (PMC8745520; doi:10.3390/ijms23010299)
Supplement: Supplementary file 1 [file ijms-23-00299-s001.zip › ijms-1508602-supplementary files/Supplemenatry Table S1.pdf]

Table S1: The clonal complex, *spa* type, MLST types, SCC*mec* types and virulence genes in six MRSA strains used in this study.

**Strain ID**	**CC**[a]	***spa* type**	**MLST (SCC*mec* type)**	**Virulence genes**
CUHK_M20_01	CC30	t019	ST30 IVc	*cap8A, cap8C, cap8G, cap8K, cap8O, chp, esaA, essA, essB, esxA, hld, hlgA, icaA, icaB, icaR, isdE, isdF, isdG, lukF-PV, lukS-PV, sak, scn, sspC,*
CUHK_M20_02	NT[b]	t437	ST59 IVa	*aur,cap8C, cap8E, cap8G, cap8I, cap8P, chp, esaB, essA, essB, esxA, hld ,hlgA, hlgB, hly/hla, icaA, icaB, icaC, icaD, icaR, isdE, srtB, lukF-PV, lukS-PV, sak, scn*
CUHK_M20_03	CC22	t3212	ST22 IV	*cap8C, cap8D, cap8E, cap8G, cap8L, cap8M, cap8P, essA, esxA, Hld, hlgA, icaA, icaB, icaD, icaR, isdC, isdE, isdG, sak, scn, srtB, sspB sspC*
CUHK_M20_04	CC45	t1081	ST45 V[c]	*cap8C, cap8G, cap8P, chp, esaB, essA, essB, esxA, esxB, hld, hlgA, icaA, icaB, icaD, isdE, sak, scn*
CUHK_M20_05	CC8	t037	ST239 III[d]	*cap8A, cap8C, cap8G cap8O, cap8K esaA, essA, essB, esxA, hld, hlgA, hlgB, hlgC, hly/hla, icaA, icaB, icaR, isdA,isdB, isdC, isdD, isdE, isdF, isdG map, sak, scn sea srtB, sspC, vWbp*

CUHK_M20_15	CC8	t008	ST8 (IVa)	*aur*, *cap8A*, *cap8C*, *cap8D*, *cap8E*, *cap8F*, *cap8G*, *cap8L*, *cap8M*, *cap8P*, *chp*, *esaA*, *esaB*, *essA*, *essB*, *esxA*, *esxB*, *geh*, *hld*, *hlgA hlgB*, *hlgC*, *hly/hla*, , *icaA*, *icaB*, *icaC*, *icaD*, *icaR*, *isdA*, *isdB*, *isdC*, *isdD*, *isdE*, *isdF*, *isdG*, *lip*, *lukF-PV*, *lukS-PV*, *map*, *sak*, *scn*, *srtB*, *sspB*, *sspC*, *vWbp*

[a]CC: Clonal Complex

[b] NT: Not typeable.

V[c]:This SCCmec_type_V does not carry a *ccrC*1, class-C2-*mec*, *ccrC1* .

III[d]: This SCCmec_type_III carries an additional class 5 *ccr*
