# Supplementary material for: Comparative Study of Two-Dimensional (2D) vs. Three-Dimensional (3D) Organotypic Kertatinocyte-Fibroblast Skin Models for Staphylococcus aureus (MRSA) Infection
Source: Int J Mol Sci. 2021 Dec 28;23(1):299. doi: 10.3390/ijms23010299 (PMC8745520; doi:10.3390/ijms23010299)
Supplement: Supplementary file 1 [file ijms-23-00299-s001.zip › ijms-1508602-supplementary files/Supporting Data S1.pdf]

Method

Solid Phase Fibrinogen and Fibronectin Binding Assay

The bacterial adherence to fibrinogen and fibronectin was studied as previously described (Wang et al., 2015; Ahmed et al., 2001). Fibrinogen (Sigma) coated plates were prepared by coating Nunc MaxiSorp 96-well plates overnight with 100µl of a 10 µg/ml bovine fibrinogen (Fg) at 4◦ C. Nunc MaxiSorp 96-well plates were coated overnight at 4°C with 100 µl of 0.02% sodium carbonate (pH 9.6) containing fibronectin (Fb) derived from human plasma (Sigma) at the concentration 10µg/ml. The plates were blocked with 2mg/ml BSA (Sigma) for 2hrs at 37°C. Exponential phase (OD_{595nm}=0.4,) bacterial cells were harvested by centrifugation at 3000g for 5mins, washed twice with Phosphate Buffered Saline (PBS) and resuspended in PBS to an OD_{595nm} of 1.0. Hundred microliters of bacterial cell suspension was added to each well and incubated for 2hrs at 37°C. The cell suspension was aspirated and the adherent bacterial cells were fixed with 25% (v/v) formaldehyde for 30 mins. After washing with PBS 100µl crystal violet dye (0.5%, w/v) was added and incubated for 10 mins. The plates were washed with PBS and air dried. The absorbance of the plates were subsequently read at 595 nm DTX 880 plate reader (Molecular Devices, CA, and USA) (Hartford et al., 1997).

Result

Solid Phase Fibrinogen and Fibronectin Binding Assay

Significant difference of adhesion of MRSA strains to the solid phase fibrinogen **(Supplementary Figure 2A)**, expressed in absorbance, was observed between the groups upon determination by One-way ANOVA, ***p<0.001. A Tukey post-hoc test revealed that the strains ST59 (1.40 ± 0.21) and ST45 (1.29 ± 0.27) exhibited maximum binding to the solid phase fibrinogen with apparent no significant statistical difference observed (p=0.75). The lowest binding to the fibrinogen was observed in ST8 (0.66 ± 0.1) and ST30 (0.767±0.1), p=0.350. ST22 (0.89 ± 0.12) and ST239 (0.95 ± 0.04) exhibited an intermediate efficiency in binding to the fibrinogen. ST22 and ST239 (p=0.93) exhibited similar binding to fibrinogen. No significant difference was observed upon comparison of ST22 to ST30 (p=0.46) and ST239 to ST30 (p=0.08). One-way ANOVA, **p<0.01 among the strains binding to the solid phase fibronectin showed significant difference **(Supplementary Figure 2B).** A Tukey post-hoc test comparing the mean of each strain with the mean of every other strain revealed that all the five strains ST8, ST30, ST59, ST45 and ST22 exhibited similar binding to the solid phase fibronectin with apparent no significant statistical difference observed among them. The lowest binding was exhibited by ST239 with significant difference between ST8 (p<0.001), ST30 (p<0.5), ST59 (p<0.01) and ST22 (p<0.05). No

significant difference was observed between ST45 and ST239 (p=0.68).

References:

Ahmed, S., Meghji, S., Williams, R.J., Henderson, B., Brock, J.H. et al. (2001). *Staphylococcus aureus* fibronectin binding proteins are essential for internalization by osteoblasts but do not account for differences in intracellular levels of bacteria. Infect Immun. *69*(5), 2872-2877.

Hartford, O., Francois, P., Vaudaux, P. and Foster, T.J. (1997). The dipeptide repeat region of the fibrinogen-binding protein (clumping factor) is required for functional expression of the fibrinogen-binding domain on the Staphylococcus aureus cell surface. *Mol. Microbiol. 25*(6), 1065-1076. doi:10.1046/j.1365-2958.1997.5291896.x

Wang, L., Bi, C., Cai, H., Liu, B., Zhong, X., et al. (2015). The therapeutic effect of chlorogenic acid against Staphylococcus aureus infection through sortase A inhibition. *Front Microbiol.*, *6*, 1031. doi:10.3389/fmicb.2015.01031
